# Supplementary material for: CooccurrenceAffinity: An R package for computing a novel metric of affinity in co-occurrence data that corrects for pervasive errors in traditional indices
Source: PLoS One. 2025 Jan 16;20(1):e0316650. doi: 10.1371/journal.pone.0316650 (PMC11737770; doi:10.1371/journal.pone.0316650)
Supplement: Attachment — Submitted filename: response to reviewers v2.4.pdf [file pone.0316650.s001.pdf]

PONE-D-24-38304
CooccurrenceAffinity: An R package for computing a novel metric of affinity in co-occurrence data that corrects for pervasive errors in traditional indices
PLOS ONE

Dear Dr. Mainali,

Thank you for submitting your manuscript to PLOS ONE. After careful consideration, we feel that it has merit but does not fully meet PLOS ONE's publication criteria as it currently stands. Therefore, we invite you to submit a revised version of the manuscript that addresses the points raised during the review process.

Please submit your revised manuscript by Nov 18 2024 11:59PM. If you will need more time than this to complete your revisions, please reply to this message or contact the journal office at plosone@plos.org. When you're ready to submit your revision, log on to https://www.editorialmanager.com/pone/ and select the 'Submissions Needing Revision' folder to locate your manuscript file.

Please include the following items when submitting your revised manuscript:

- A rebuttal letter that responds to each point raised by the academic editor and reviewer(s). You should upload this letter as a separate file labeled 'Response to Reviewers'.
- A marked-up copy of your manuscript that highlights changes made to the original version. You should upload this as a separate file labeled 'Revised Manuscript with Track Changes'.
- An unmarked version of your revised paper without tracked changes. You should upload this as a separate file labeled 'Manuscript'.

If you would like to make changes to your financial disclosure, please include your updated statement in your cover letter. Guidelines for resubmitting your figure files are available below the reviewer comments at the end of this letter.

If applicable, we recommend that you deposit your laboratory protocols in protocols.io to enhance the reproducibility of your results. Protocols.io assigns your protocol its own identifier (DOI) so that it can be cited independently in the future. For instructions see: https://journals.plos.org/plosone/s/submission-guidelines#loc-laboratory-protocols. Additionally, PLOS ONE offers an option for publishing peer-reviewed Lab Protocol articles, which describe protocols hosted on protocols.io. Read more information on sharing protocols at https://plos.org/protocols?utm_medium=editorial-email&utm_source=authorletters&utm_campaign=protocols.

We look forward to receiving your revised manuscript.
Kind regards,
Abhik Ghosh
Academic Editor
PLOS ONE
Journal Requirements:

When submitting your revision, we need you to address these additional requirements.

1. Please ensure that your manuscript meets PLOS ONE's style requirements, including those for file naming. The PLOS ONE style templates can be found at https://journals.plos.org/plosone/s/file?id=wjVg/PLOSOne_formatting_sample_main_body.pdf and https://journals.plos.org/plosone/s/file?id=ba62/PLOSOne_formatting_sample_title_authors_affiliations.pdf

2. Please note that PLOS ONE has specific guidelines on code sharing for submissions in which author-generated code underpins the findings in the manuscript. In these cases, we expect all author-generated code to be made available without restrictions upon publication of the work. Please review our guidelines at https://journals.plos.org/plosone/s/materials-and-software-sharing#loc-sharing-code and ensure that your code is shared in a way that follows best practice and facilitates reproducibility and reuse.

> AUTHORS' RESPONSE:
> All figures in the manuscript developed with code are fully and exactly reproducible with the supplied R code or with the GitHub repo indicated in the manuscript. Figs 1-3 were developed by drawing them directly. Figs 4-6 and 9 – in the final form as seen on the manuscript – were developed using the R package functions. The exact code for these figures is provided in the figure or the caption. R code for Figs 7-8 – again, as presented in the manuscript – is available at the following GitHub repo: https://github.com/kpmainali/CooccurrenceAffinity_PublicationScript
>
> The package is live on CRAN at <https://cran.r-project.org/web/packages/CooccurrenceAffinity/index.html> and at GitHub at https://github.com/kpmainali/CooccurrenceAffinity.

3. Please note that funding information should not appear in any section or other areas of your manuscript. We will only publish funding information present in the Funding Statement section of the online submission form. Please remove any funding-related text from the manuscript.

> AUTHORS' RESPONSE:
> The funding information section from the main text has been removed. Since "Acknowledgements" carried only the funding information, the entire section of Acknowledgements has been eliminated now.

4. Thank you for stating the following financial disclosure: "KM was supported by the Grayce B. Kerr Fund, Inc, and by the National Science Foundation DBI-1639145 under funding received for the National Socio-Environmental Synthesis Center (SESYNC)."
Please state what role the funders took in the study. If the funders had no role, please state: "The funders had no role in study design, data collection and analysis, decision to publish, or preparation of the manuscript." If this statement is not correct you must amend it as needed. Please include this amended Role of Funder statement in your cover letter; we will change the online submission form on your behalf.

AUTHORS' RESPONSE:
We have now added the requested details in our cover letter. Thank you for taking care of updating our online submission form.

5. Please provide a complete Data Availability Statement in the submission form, ensuring you include all necessary access information or a reason for why you are unable to make your data freely accessible. If your research concerns only data provided within your submission, please write "All data are in the manuscript and/or supporting information files" as your Data Availability Statement.

AUTHORS' RESPONSE:
We have provided an accurate description of data availability in the online submission form now. This statement reads as: "Several of the figures were developed based on hypothetical data, which is provided in the manuscript in the figure or figure caption. One figure (Fig 6) relies on a freely available data of Darwin's finches that can be loaded from another package "cooccur"."

6. Please amend the manuscript submission data (via Edit Submission) to include author Eric Slud.

AUTHORS' RESPONSE:
Author's profile is now created for Eric Slud.

Reviewers' comments:

Reviewer's Responses to Questions
Comments to the Author

1. Is the manuscript technically sound, and do the data support the conclusions?

The manuscript must describe a technically sound piece of scientific research with data that supports the conclusions. Experiments must have been conducted rigorously, with appropriate controls, replication, and sample sizes. The conclusions must be drawn appropriately based on the data presented.
Reviewer #1: Partly
Reviewer #2: Yes

---

2. Has the statistical analysis been performed appropriately and rigorously?
Reviewer #1: Yes
Reviewer #2: Yes

---

3. Have the authors made all data underlying the findings in their manuscript fully available?

The PLOS Data policy requires authors to make all data underlying the findings described in their manuscript fully available without restriction, with rare exception (please refer to the Data Availability Statement in the manuscript PDF file). The data should be provided as part of the

manuscript or its supporting information, or deposited to a public repository. For example, in addition to summary statistics, the data points behind means, medians and variance measures should be available. If there are restrictions on publicly sharing data—e.g. participant privacy or use of data from a third party—those must be specified.
Reviewer #1: Yes
Reviewer #2: Yes

---

4. Is the manuscript presented in an intelligible fashion and written in standard English?

PLOS ONE does not copyedit accepted manuscripts, so the language in submitted articles must be clear, correct, and unambiguous. Any typographical or grammatical errors should be corrected at revision, so please note any specific errors here.
Reviewer #1: No
Reviewer #2: Yes

---

5. Review Comments to the Author

Please use the space provided to explain your answers to the questions above. You may also include additional comments for the author, including concerns about dual publication, research ethics, or publication ethics. (Please upload your review as an attachment if it exceeds 20,000 characters)

Reviewer #1:

Manuscript PONE-D-24-38304 titled "CooccurrenceAffinity: An R package for computing a novel metric of affinity in co- occurrence data that corrects for pervasive errors in traditional indices" presents a new R package – CooccurrenceAffinity. This package can be used to analyze species co-occurrence data an represent them using graphical visualization. To this end, the authors use a new metric – alpha, assuming null distribution or randomization of the association, thus adding to the accuracy of the analysis.

Here are my thoughts.

Try and avoid repetitions of phrases between title and keywords.

> AUTHORS' RESPONSE:
> Thank you. We indeed have redundant words in keywords. We have now eliminated the repetitions from title.

Line 18 – please elaborate the phrase “many problems” in about a line. You may wish to combine part of the trailing line as well.

AUTHORS’ RESPONSE:
We have now revised the text to indicate what the problems are. The revised text reads as: “Analysis of co-occurrence data in ecology and biogeography with traditional indices has led to many problems such as sensitivity of the indices to prevalence and the same value representing either a strong positive or strong negative association across different datasets.” The following sentence of the text also clarifies that the two key problems were sensitivity to prevalences and failure to provide similar index values for distinct species-pairs with similar degrees of association.

Minor grammatical errors relating to verb and article use are present in the manuscript. For example, line 30 among others.

AUTHORS’ RESPONSE:
We have now carefully revised the paper to maintain proper subject-verb agreement and exercised greater care in specifying referents for adjectival clauses.

The major concern with this manuscript is that the authors have chosen to use a very confusing approach towards their main goal. The index as proposed by the authors and claimed to be free from errors unlike traditional methods.

AUTHORS’ RESPONSE:
This software package is certainly non-standard because it presents conceptual advances about various quantities, and four types of confidence intervals including a comparison of their coverage probability. Including all of these in one paper has probably made the manuscript challenging to navigate, given its breadth. Our manuscript has multiple goals: (i) moving past prevalence-dependent indices; (ii) enabling pairwise co-occurrence analysis -- estimates and confidence intervals -- in settings where there might be strong positive or negative associations; and (iii) providing a conceptual model and an interpretable estimation target so that multiple species pairs could be appropriately clustered on the basis of similarity of their alpha estimates. We have done our best in breaking down various components into separate sections and have used illustrations extensively. The goal of the package is to provide statistical tools for point and interval estimation of a target, the affinity parameter α, where past practice in Ecology has largely been focused only on testing the null hypothesis that $\alpha = 0$.

The proposed affinity index relies on assumptions similar to the fixed row-equiprobable column (FE) null model, which is not mentioned. This makes the claims of the authors regarding novelty as overestimated, as the method is not as original as is led to believe.

AUTHORS' RESPONSE:

The criticism is unfair, because the novelty is not to move away from the FE setting but rather to provide a meaningful non-null parameter value quantifying strength of association in that setting. FE model does not offer a solution to compute a statistic measuring strength of association in non-null settings. FE model allows one to compute the probability of observing the occurrences/co-occurrences given the null model.

Although we offered a concise response to the reviewer's comment above, we now provide a more comprehensive historical account of co-occurrence analysis and our contribution's specific role within it.

We need to understand FE framework first before we decide what is an important contribution in the area of precise null model and a reliable estimate of association. The FE and FF approaches for randomizing a 2x2 matrix (see our Fig 1a) have been widely discussed and utilized in ecological and biogeographical literature over the past few decades. Without a clear understanding of the null model's structure, researchers who developed these approaches relied on randomizing the components of a 2x2 matrix through multiple iterations, examining how the observed data matrix aligns with the empirical null. In a species-by-site matrix of presence/absence, FE (fixed–equiprobable) assumes species prevalence is fixed during randomization. This approach does not guarantee that the number of species at a given site is fixed. Conversely, FF (fixed-fixed) assumes both row and column sums are fixed during randomization. In this method, when a randomization algorithm selects a fixed number of sites for a species, it forces another species to occupy certain sites, ensuring the total number of species at a site remains constant.

A key contribution of these two randomization approaches is recognizing that different situations in community ecology suit either the FE or FF approach while developing an empirical null distribution. It is important to note that FE and FF approaches only address assumptions about null distribution, specifically what aspects of a species-site matrix should remain constant during randomization for simulating an empirical null distribution. Veech (Veech, 2013) summarized these approaches succinctly: "These two classes of randomization were given the labels of fixed–fixed (F–F) and fixed–equiprobable (F–E) by Gotelli & Ellison (2002a)."

It is crucial to acknowledge that neither approach identified the mathematical structure of the null model. The first study in ecological literature to correctly identify the null distribution was (Veech, 2013) which assumed the long form of equation as a solution of the null model did not have a distribution. Veech stated: "In this paper, I develop and present a distribution-free and metric-free approach to analysing species co-occurrence patterns." In a subsequent paper, his team realized the previously identified null

distribution followed a hypergeometric distribution (Griffith, Veech, Marsh, & others, 2016). These seminal studies by Veech introduced the correct form of the null model in ecological literature, specifically in a species-site occupancy problems, even though the null model for cooccurrence problem was recognized for about a century.

What did not occur until our publication of alpha hat in 2022 (Mainali, Slud, Singer, & Fagan, 2022) was the development of a reliable estimate of association. To compare a null model with an estimate, consider the analogy of an unfair coin. An unfair coin has a probability of heads that differs from 0.5. With a sufficiently large sample size, one could conclude the coin is unfair if the p-value is less than assumed alpha (e.g., < 0.05). However, this conclusion provides no information about the extent of unfairness. There can be varying degrees of unfairness, but a p-value cannot differentiate between them for several reasons discussed in (Mainali & Slud, 2024) including the fact that P-value is a function of sample size. Another example showing the significance of an estimate is determining that an individual's body weight significantly differs from the population's weight based on p-value, yet having no indication of how different it is. Statistical significance only shows evidence of a difference, without addressing the magnitude of the difference. A Z-score in this example can be used to estimate how extreme a value is.

We made an important advancement in this area by developing the MLE of alpha in 2022. Our Science Advances paper provided adequate historical context, crediting Fisher, Huygens, and Pearson for developing the original tools and Veech and Griffith for introducing the null model to ecological literature. From our paper:

> "This distribution was known to Huygens as early as 1657 in a gambling context (Huygens, 1714), and was proposed as the basis for Fisher's (Fisher, 1934) famous 'exact test' for independence of row and column categories in a 2×2 contingency table, following Pearson's (Pearson, 1900) earlier use of the χ^2 large-sample test of the same hypothesis. Recent work introduced the hypergeometric as a null distribution in ecology (Griffith et al., 2016; Veech, 2013)."

We did not cite the FE model in this manuscript for two reasons: First, the reviewer's comment says: "The proposed affinity index relies on assumptions similar to the fixed row-equiprobable column (FE) null model, which is not mentioned." We do not take "assumptions" as an item of novelty. Alpha hat and the FE model both use the same set of assumptions. Beyond assumptions, we provide a full statistical model with the parameter quantifying degree of associations, and that is new in ecological and biogeographical literature. FE model, unfortunately, does not provide a measure of association; see Gotelli (2000) and Wright et al. (1998). In our paper, we (Mainali et al., 2022) provided four "classic assumptions" that are precisely stated and imply the hypergeometric null hypothesis under fixed marginals for a co-occurrence table. Second, our parameter and software apply to any type of co-occurrence data, well beyond ecology and biogeography, let alone species-site matrix. Citing FE model from a narrowly focused discipline would not do justice to the broader applicability of our work.

The second critique in my opinion is the assumption that all sites are equally suitable for species, which may not hold in real-world datasets with heterogeneous sites, such as islands or even populations dispersed along gradients. The proposed method may underperform in such cases. The assumption of equiprobability needs to be revisited and established with proper justification i.e., limitations of the presented theory.

AUTHORS' RESPONSE:

This comment relates to the fact that our Affinity null model assumes that all sites are equally suitable for each species considered separately. This was also a comment in a commentary (Ulrich, Sfenthourakis, Strona, & Gotelli, 2022) published criticizing our affinity model. We agree with the reviewer that it would be desirable to extend the assumptions and make these tools applicable to another setting (called FF model), after developing [what would again be completely new] a parameter in the FF settings quantifying non-null association between species. That is on our research agenda, but no one has done it yet.

However, it is important to note that a large community of ecologists and biogeographers continue to use null-model *P*-values and standardized indices which precisely utilize a hypergeometric null model; Veech's software (Griffith et al., 2016) directly implements this hypergeometric null. Both the hypergeometric null and the traditional randomization of a 2x2 matrix assume the exchangeability as the FE model. Our Affinity model offers significant improvements over the FE model and standardized co-occurrence indices. In our recently published commentary (Mainali & Slud, 2024), we show how unreliable it is to use p-values as estimates of association (see section 1 for detailed analysis), and Figure 2c,d and the associated text demonstrate how standardization is unreliable and inappropriate based on statistical theory.

Site exchangeability is a feature of our model because it was developed for a wide range of co-occurrence data scenarios (including genetics, neurobiology, sociology, ecology, etc. as demonstrated by the citations of the Affinity model paper in these fields) with specific null model assumptions that are appropriate for many scenarios. *More importantly, we do not understand why this comment is being raised in the context of this software paper. This is not a scientific paper; it focuses on the software and its applicability, not on advancing a specific scientific hypothesis.*

My third concern about the R package's implementation is inconsistent results from the same data. The reliability of the package is brought into question. This limits practical applicability.

AUTHORS' RESPONSE:
Please provide a screenshot, along with the code, to show that this is the problem. We have not experienced this issue since the package was fully developed. Interestingly, the reviewer's comment seems to echo a negative commentary by (Ulrich et al., 2022), which accused our package of producing inconsistent outputs without providing any evidence.

Our response (Mainali & Slud, 2024) to this commentary went through two rounds of peer review, yet the editors were unable to offer any proof of inconsistent results.

For transparency, we have provided a timeline of significant developments on our GitHub at https://github.com/kpmainali/CooccurrenceAffinity. Shortly after the package's initial release in December 2021 with a few basic functions, we added several useful functions. During this process, one function, ML.Alpha(), was affected for a brief period. This issue was promptly resolved. As indicated in the timeline, we included the following note:

> Mar 18, 2022: An error was inadvertently introduced in ML.Alpha() in early March that affected computation of upper bound of Alpha MLE. The error was fixed on March 18, 2022. CooccurrenceAffinity package installed prior to March 18, 2022 should be removed and a new version currently available should be reinstalled for it to function properly.
>
> Since then, we have never observed an inconsistent model output.

Reviewer #2:

The manuscript titled "CooccurrenceAffinity: An R package for computing a novel metric of affinity in co-occurrence data" introduces a valuable tool for ecological and biogeographical research. The CooccurrenceAffinity R package offers a new affinity metric, α, addressing the limitations of traditional co-occurrence indices like Jaccard and Sørensen-Dice. The package's ability to compute maximum likelihood estimates (MLE) of α, along with four different confidence intervals, makes it a powerful tool for researchers dealing with binary presence-absence data. The manuscript is well-structured and provides detailed explanations of the methods and the theoretical underpinnings of the metric. The integration of this package into ecological workflows can provide more reliable interpretations of species associations, which is a significant contribution to the field. However, some areas need clarification and improvement, both in terms of mathematical rigor and writing style, to enhance the paper's clarity and utility for readers.

Comment1:
The manuscript heavily relies on MLE for α, which can lead to biased results in small sample sizes, a common issue in ecological studies. It would be beneficial to include a discussion on the limitations of MLE in such contexts. Consider exploring alternative methods, such as bias-corrected MLE or Bayesian estimators, which may offer more robust results when working with small datasets. Adding such a discussion would improve the paper's applicability across a wider range of sample sizes.

AUTHORS' RESPONSE:
We thank the reviewer for the positive comments about the value of the tools we provide. We cannot disagree with this comment that Maximum Likelihood Estimators in small samples often turn out to be slightly biased. But given that the Ecology community has had no agreed target parameter for estimation, we do not know what the reviewer has in mind as an alternative to our MLE. (Debiasing procedures based on jackknife or bootstrap may not be very reliable in small data-samples, so until alternative estimators with less bias are proposed, we have no immediate way to resolve this issue.) For reporting results about the

α parameter based on small-sample data, we recommend that the degree of association would be best summarized by a Confidence Interval. But we think Ecologists would actually use the MLE not as an absolute measure of anything, but rather as a tool for grouping or clustering species-pairs with similar degrees of association by means of their similar alpha-hat MLEs. We have shown elsewhere (Mainali & Slud, 2024) that the MLEs do reliably center near the correct α in simulated settings where α is known. So we think that in that sense, the MLE is fit for the purpose it will be put to.

Comment2:
The use of hat α values to avoid ±∞ for extreme co-occurrence values introduces potential distortions in the results. It's important to further explain the impact of these hat values on interpretation and provide clear guidance for users on how to handle such cases in biological applications.

AUTHORS' RESPONSE:
We have now expanded the section "Special handling of the most extreme cooccurrences" providing clearer guidelines. We now explain, although perhaps still too briefly, that the fault involved in truncating an infinite log-odds-ratio MLE to $\log(2N^2)$, which for N of order 100 is roughly 10 (still a phenomenally large odds ratio), will not distort the grouping of very strongly associated species-pairs and contrasting them with less strongly associated pairs. The problem with these infinite or near-infinite α values is not with the model or estimate, but rather with the extremely poor resolution of the semi-infinite intervals of α values compatible with nested 2x2 table data. Because of this poor resolution, we strongly prefer to report degrees of association in terms of confidence intervals (one-sided in the case of nested tables) rather than point estimates when data sizes are small or cooccurrence values are extreme.

Comment3:
While the manuscript presents four types of confidence intervals, it does not fully explain the trade-offs between more conservative intervals (e.g., Clopper-Pearson) and exploratory ones (e.g., midP, midQ). Including a more detailed discussion on when to use each type of CI, especially in exploratory versus confirmatory research, would enhance the utility of the method. Guidance on managing undercoverage risks in exploratory studies would also be helpful.

AUTHORS' RESPONSE:
We thought that our two sections under the headings "Preference for Conservative or Close-to-nominal-coverage Intervals?" and "Good practice" provided a useful discussion about the alternative exploratory versus confirmatory settings where respectively near-nominal or conservative confidence-interval coverage would be preferred. The trade-off between intervals that have conservative versus near-nominal coverage is related to interval length. That is why we have taken pains to recommend the shortest intervals available, Blaker instead of CP-type among conservative intervals. We have stated that the midP intervals tend to lie within the Blaker and CP-type intervals. We think that the choice among these types of intervals will be made according to the necessity of ensuring at-least-nominal coverage. Finally, users of our software and ideas will see clearly that the Blaker

intervals are shorter than the Clopper-Pearson and in that sense more desirable, while there is nothing to choose between the midP and midQ intervals, which are essentially identical in practice.

To make this concept more accessible to practitioners, we have substantially revised the "Good practice" section, along the lines of this Response to the Reviewer's comment.

Comment4:
The paper overlooks the need for adjusting for multiple comparisons when analyzing numerous species pairs, which can increase the risk of Type I errors. Addressing this by suggesting corrections such as Bonferroni or False Discovery Rate (FDR) would enhance the reliability of the results in large-scale ecological analyses, where multiple pairwise comparisons are common.

AUTHORS' RESPONSE:
From a statistician's point of view, multiple comparisons adjustments such as Bonferroni are used when there is an overall interest in whether any of a number of possibly related significance tests would reject the null hypothesis. This analysis setting arises when a statistician would report any rejection as evidence that some real phenomenon was occurring. Contrast this with a setting where many parallel hypotheses tests are done with the idea that some are likely to be significant and we want to report and group the ones that are. We can imagine Ecology settings in which an experiment-wise test across many species-pairs might be used to check a null hypothesis of no association in any of the pairs, and in that case the reviewer's suggestion is relevant. But much more commonly, and in all the Ecology settings we are aware of in published literature, ecologists looking at multiple species-pairs are mostly interested in screening those species-pairs with non-null association and in clustering species-pairs by similarity of degree of association, and not so much in an experiment-wise test of whether there are any species-pairs showing association. For that reason, our judgment was that the use-cases for the tools we provide would seldom require multiple-comparisons adjustments.

A Bonferroni correction to achieve a specified experiment-wise error rate would generally have the effect of making it difficult to establish non-null association in species-pairs. So we do not advocate those. On the other hand, in Ecology as in other biological settings such as genomics where many entities are screened, there could be interest in a False-Discovery-Rate analysis of which non-null species pairs would best be studied in detail. However, such a setting is still not common in Ecology, and we thought and still do think that covering this topic is beyond the scope of our software applications.

There are several ways to address these problems, and users can select the right approach for their needs. For example, there are a number of R packages that offer solutions. Users can simply pass the vector of all p-values from repeated tests to p.adjust() in base R and obtain results based on their preferences. We do not need to reinvent the wheel here. Furthermore, as this is a software paper, we do not need to delve into broader related statistical theories.

Comment5:
The assumption of independent co-occurrences ignores spatial autocorrelation, which is crucial in ecological studies. Acknowledging this limitation and suggesting future extensions to incorporate spatial dependence would make the method more broadly applicable to real-world scenarios where spatial structure plays a significant role.

AUTHORS' RESPONSE:
This comment raises a valid point. Typical inferential statistics assume independent and identically distributed (iid) observations, which is a common assumption in many models. Addressing spatial dependence requires specialized approaches that are beyond the scope of this project. It would be worthwhile to undertake a modeling effort to account for species-pair affinities by means of regression models in which the applicable alpha at a specific site is functionally related to covariates at that site, some of which would be spatial coordinates. Our software focuses on its core functionality, leaving the management of spatial autocorrelation to future research projects.

Comment6:
The ecological interpretation of α as a log-odds ratio may not be intuitive for all readers. Providing additional examples or practical explanations of what different values of α represent in terms of species interactions (e.g., competition, facilitation) would make the metric more accessible and meaningful for ecologists.

AUTHORS' RESPONSE:
Thank you for your suggestion. With ecologists and biogeographers in mind, we created Fig. 2, where, in steps a-c, we visually demonstrate a case of species affinity by placing them in various sites. We then rearrange the sites to align with the "balls in boxes" illustration. This progression naturally introduces the definition of alpha as a log-odds ratio, with the preference parameters p1 and p2 already integrated into the visual representation of species interactions. This approach provides an intuitive lead-in to the mathematical concept of alpha.

We think the appropriate additional exposition of the usefulness of odds ratios in categorical data analysis is covered both in (Agresti, 2013) and the more elementary book (Agresti, 2018).

Comment7:
Some sentences in the manuscript are overly long and complex, making them difficult to follow. Simplifying these sentences and using clearer, more concise language will improve the readability and overall flow of the manuscript.

AUTHORS' RESPONSE:
We have attempted to streamline and simplify the writing wherever possible.

Minor

Comment1:
The manuscript uses passive voice excessively, which can make the writing less engaging. For instance, "It was found that the MLE provided reliable estimates" could be improved by switching to active voice: "We found that the MLE provided reliable estimates." This would make the writing clearer and more direct.

> AUTHORS' RESPONSE:
> We have eliminated a very large number of uses of passive voice. While some remain, we believe the style and flow of the manuscript are much improved. We hope the reviewer agrees.

Comment2:
Some terms are not clearly defined when first introduced. For example, "Affinity is calculated using the log-odds ratio" is unclear without defining what affinity or the log-odds ratio means. A clearer explanation would help, such as: "Affinity refers to the degree of association between two species based on their co-occurrence. The log-odds ratio quantifies this relationship, indicating how much more likely species are to co-occur than by chance."

> AUTHORS' RESPONSE:
> We presented the definition of α in the section "The parameter" and only then introduced the log odds terminology. So we think that we did not leave terms undefined as claimed here.

Comment3:
Some sentences are too long and include multiple ideas, making them difficult to follow. For example: "The novel metric of affinity, α, is introduced to resolve issues in traditional indices, and it is computed through the MLE, which provides a better estimation for species association, making it more reliable for large datasets, although small datasets may still present challenges." This could be broken down into clearer sentences: "The novel metric of affinity, α, resolves issues in traditional indices. It is computed using the MLE, providing a more reliable estimation for species association in large datasets. However, small datasets may still present challenges."

> AUTHORS' RESPONSE:
> We acknowledge that our sentences were too long in places, to the detriment of clarity. We have revised, reworded and shortened sentences where we found such deficiencies of style.

Comment4:
The abstract is wordy and lacks a clear focus on the paper's key contributions. For example, "We provide functions for analysis and plotting based on various data formats, and compute the novel metric along with traditional indices such as Jaccard and Simpson." This could be made more concise, such as: "We introduce the CooccurrenceAffinity R package, which computes a novel metric of species affinity (α) and corrects biases in traditional indices. The package provides functions for analyzing co-occurrence data and generating visual outputs."

AUTHORS' RESPONSE:
We reworded the Abstract to explain better what were the "many problems" that we think we have resolved.

6. PLOS authors have the option to publish the peer review history of their article (what does this mean?). If published, this will include your full peer review and any attached files.

If you choose "no", your identity will remain anonymous but your review may still be made public.

AUTHORS' RESPONSE: Okay to publish.

Do you want your identity to be public for this peer review? For information about this choice, including consent withdrawal, please see our Privacy Policy.
Reviewer #1: No
Reviewer #2: No

REFERENCES

Agresti, A. (2013). *Categorical Data Analysis* (3rd ed.). John Wiley & Sons, Inc., New Jersey.

Agresti, A. (2018). *An Introduction to Categorical Data Analysis*. John Wiley & Sons.

Fisher, R. A. (1934). *Statistical Methods for Research Workers* (5th ed.). Oliver and Boyd, Edinburgh and London.

Gotelli, N. J. (2000). Null model analysis of species co-occurrence patterns. *Ecology*, *81*(9), 2606–2621.

Griffith, D. M., Veech, J. A., Marsh, C. J., & others. (2016). Cooccur: Probabilistic species co-occurrence analysis in R. *Journal of Statistical Software*, *69*(2), 1–17.

Huygens, C. (1714). *Libellus de Ratiociniis in Ludo Aleae. Or, The value of all chances in games of fortune; cards, dice, wagers, lotteries, &c. mathematically demonstrated*. S. Keimer, London.

Mainali, K. P., & Slud, E. (2024). Null model analyses are not adequate to summarize strong associations: Rebuttal to Ulrich et al.(2022). *Journal of Biogeography*, *51*, 263–268.

Mainali, K. P., Slud, E., Singer, M. C., & Fagan, W. F. (2022). A better index for analysis of co-occurrence and similarity. *Science Advances*, *8*(4), 1–9.

Pearson, K. (1900). On the criterion that a given system of deviations from the probable in the case of a correlated system of variables is such that it can be reasonably supposed to have arisen from random sampling. *Philosophical Magazine*, *50*(302), 157–175.

Ulrich, W., Sfenthourakis, S., Strona, G., & Gotelli, N. J. (2022). Simple null model analysis subsumes a new species co-occurrence index: A comment on Mainali et al. (2022). *Journal of Biogeography*, *49*, 2321–2324.

Veech, J. A. (2013). A probabilistic model for analysing species co-occurrence. *Global Ecology and Biogeography*, *22*(2), 252–260.

Wright, D. H., Patterson, B. D., Mikkelson, G. M., Cutler, A., & Atmar, W. (1998). A comparative analysis of nested subset patterns of species composition. *Oecologia*, *113*(1), 1–20.
